# Transcatheter arterial chemoembolization after stopping sorafenib therapy for advanced hepatocellular carcinoma

**DOI:** 10.1371/journal.pone.0188999

**Published:** 2017-11-30

**Authors:** Yao-Kuang Huang, Chieh-Ling Yen, Sz-Iuan Shiu, Shou-Wu Lee, Pi-Yi Chang, Hong-Zen Yeh, Teng-Yu Lee

**Affiliations:** 1 Division of Gastroenterology & Hepatology, Department of Internal Medicine, Taichung Veterans General Hospital, Taichung, Taiwan; 2 Department of Medicine, Chung Shan Medical University, Taichung, Taiwan; 3 Department of Radiology, Taichung Veterans General Hospital, Taichung, Taiwan; University of North Carolina at Chapel Hill School of Medicine, UNITED STATES

## Abstract

Targeted therapy is currently the standard treatment for advanced hepatocellular carcinoma (HCC), but an effective treatment after the discontinuation of sorafenib therapy remains uncertain. We aim to investigate the survival benefits of transcatheter arterial chemoembolization (TACE) after stopping sorafenib therapy. We retrospectively analyzed all patients with advanced HCC, who had received palliative TACE after terminating sorafenib therapy, from January 2008 to June 2016. Patients who were in the terminal stage (Child-Pugh class C or performance status 3–4), who received a liver transplantation, or who had received any HCC treatment other than TACE, were excluded. Finally, 28 patients were recruited as the TACE group, and were randomly matched 1:1 by age, gender, Child-Pugh class, extrahepatic metastasis, and portal vein thrombosis with 28 controls who only received supportive care. For avoiding any immortal time bias, the index date of outcome follow-up was also matched. Cumulative incidences of, and hazard ratios (HRs) for, patient mortality were analyzed. The baseline demographic data between the TACE group and the control group were similar, but the 1-year overall survival rate in the TACE group was significantly higher than that of the control group (41.2%, 95% confidence interval [CI]: 19.4–63.0% vs. 24.5%, 95% CI: 6.3–42.7%; *p* < 0.01). In multivariate analysis, after adjusting for alpha-fetoprotein > 400ng/mL, Child-Pugh class B, and tumor extension > 50% of liver volume, TACE was independently associated with a decreased mortality risk(HR 0.19, 95% CI: 0.08–0.42). In addition, tumor extension > 50% of the liver was another independent prognostic factor associated with an increased mortality risk (HR 2.99, 95% CI: 1.31–6.82). Multivariate stratified analyses verified the association of TACE with a decreased mortality rate in each patient subgroup (all HR < 1.0). By controlling intrahepatic tumor growth, TACE may be a treatment option for use in improving patient survival in advanced HCC, after the termination of sorafenib therapy.

## Introduction

Hepatocellular carcinoma (HCC) is currently the second leading cause of cancer mortality worldwide [1]. Unfortunately, approximately one third of the patients belong to the advanced stage of HCC, with an average survival period of only 6–12 months [2]. Sorafenib is currently the standard treatment for advanced stage HCC [3,4]. However, the response rate of sorafenib therapy has been reported in previous literature to be less than 5%, while the median patient survival time was prolonged for only about 3 months [3]. Moreover, as many as 30–40% of patients could not tolerate the side effects of sorafenib therapy, so it had to be discontinued early [5,6]. Thus, the majority of patients with advanced HCC who were treated with sorafenib, will eventually stopped sorafenib therapy due to either disease progression or side effect intolerance. Unfortunately, after stopping sorafenib therapy, the one-year patient survival rate was only around 30–40% [7,8]. Therefore, finding an effective treatment to improve patient survival after stopping sorafenib therapy for advanced HCC is mandatory.

Although clinical trials for second-line systemic treatment after stopping sorafenib therapy have been conducted, the overwhelming majority of studies’ results were disappointing [9–12]. Currently, according to the international practice guidelines for the management of HCC, an available rescue treatment after cessation of sorafenib therapy remains unclear [4,13]. Furthermore, although regorafenib has recently been approvedas a second-line treatment for HCC patients who have previously received sorafenib therapy [14], a wider use of the drug in the real world is still pending. It remains urgent to establish an effective, approachable and affordable alternative treatment for patients upon their terminationfrom sorafenib therapy.

Transcatheter arterial chemoembolization (TACE) is an effective local-regional treatment for HCC, where TACE can improve patient survival rates and has been recommended as the standard treatment for intermediate-stage HCC [15,16]. Moreover, TACE may also improve patient survival in select patients with advanced HCC [17,18]. Although extrahepatic metastases may exist in advanced-stage HCC, patients may still die of intrahepatic tumor progression [19]. In this way, TACE may improve survival through its control of intrahepatic tumors [19,20]. However, the evidence supporting the use of TACE for advanced HCC after stopping sorafenib therapy remains lacking. In this study, by comparing patients receiving palliative TACE with those who are not, we aimed to assess the survival benefits of TACE after patients had stopped sorafenib therapy.

## Materials and methods

### Study design

This retrospective cohort study was conducted at a tertiary referral center in central Taiwan. All patients who were diagnosed as having advanced HCC and had received sorafenib therapy were screened from January 2008 to June 2016. HCC was diagnosed through eitherpathological confirmation or typical dynamic image presentations of HCC according to the current practice guidelines [21]. The image studies were independently reviewed by two radiologists. This study was approved by the Ethics Committee of Taichung Veterans General Hospital.

### Study subjects

**Fig 1:**
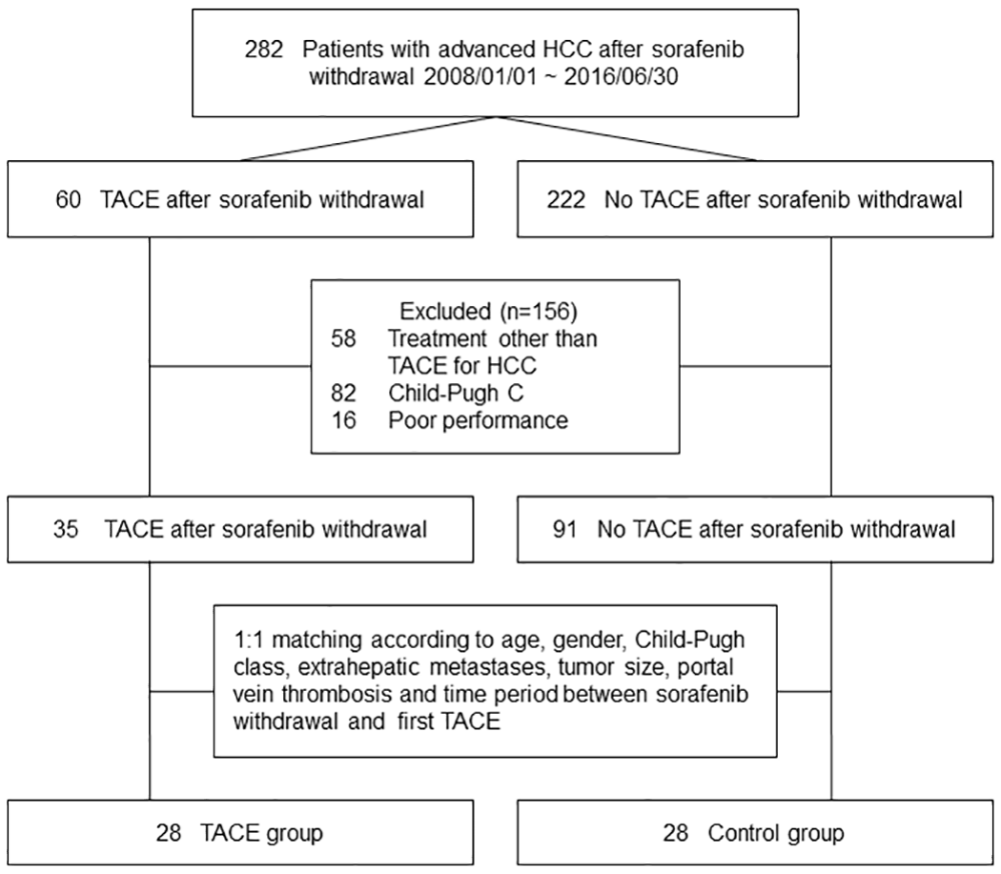
Flow algorithm of patient selection.

The flow algorithm of patient selection is shown in Fig 1. We located all 282 adult patients who were in advanced-stage HCC and had received sorafenib therapy from January 2008 to June 2016. At the time of stopping sorafenib therapy, any patients whose liver function belonged in Child-Pugh class C, and whose performance status belonged in Eastern Cooperative Oncology Group score 3–4 were excluded. In addition, the patients who had received any HCC treatment other than TACE after stopping sorafenib therapy, including radiotherapy, systemic chemotherapy or other therapies in clinical trials, were also excluded. Thus, patients were divided into two groups according to whether they had received TACE (the TACE cohort) or had not (the control cohort) after stopping sorafenib therapy. Furthermore, patients in the TACE cohort were randomly matched 1:1 with patients in the control cohort by age, gender, Child-Pugh class, extrahepatic metastasis, portal vein thrombosis, and tumor size. Eventually, 28 patients were recruited from both the TACE group and the control group for final analysis in this study.

### Patient selection for TACE treatment

According to the consensus guideline for HCC management of the Taiwan Liver Cancer Association and the Gastroenterological Society of Taiwan [22] although sorafenib is the standardsystemic therapy for patients with advanced HCC, TACE may also be considered as a locoregional treatment in patients not experiencing severe liver decompensation (Child-Pugh C). However, the survival benefits of TACE treatment after stopping sorafenib therapy remain uncertain, so only a proportion of patients in Child-Pugh A or B would be recommended for TACE.

### TACE procedure

Only conventional TACE is reimbursed by the National Health Insurance in Taiwan. Basically, the feeding arteries of HCCs were selectively catheterized for the purpose of preserving liver parenchyma, before transarterial chemotherapy was performed using a mixture of lipiodol and a chemotherapeutic agent. The feeding arteries were subsequently embolized with gel foam until complete flow stagnation was achieved.

### Main outcome measurement

Cumulative incidences of patient mortality were calculated, with the major outcome in this study being the overall survival rate over one year. The index date of outcome follow-up was the date of the first TACE in the TACE group. For the purpose of eliminating any immortal time bias, the follow-up index date of the matched control was given by matching the time period between the sorafenib stopping date and the first TACE date [23,24]. Patients were followed up until either mortality, or the end of the one-year follow-up.

### Prognostic factor assessment

Detailed data regarding potential prognostic factors were individually retrieved from the medical records of study subjects, including Child-Pugh score, Alpha-fetoprotein (AFP) levels, performance status and tumor image characteristics on the index date of outcome follow-up. In addition, data on the dosage and duration of sorafenib therapy, the reasons for sorafenib withdrawal, and the history of local-regional HCC treatment prior to sorafenib therapy were also collected.

### Statistical analysis

Continuous data was presented as median values (25%-75% interquartile ranges), and tested with Wilcoxon signed rank test. Discrete data was shown as numbers (percentages), and tested with the McNemar test. Cumulative incidences of overall mortality after the follow-up index date were calculated, and the calculated rates were expressed as the estimated number, along with the 95% confidence interval (CI). One-year overall survival rates were compared using the Kaplan-Meier’s method. After adjusting for potential confounding factors, multivariate regression analyses were conducted to determine the independent prognostic factors for overall mortality, while hazard ratios (HRs) were determined by Cox proportional hazard models. Multivariate stratified analysis for the effect of TACE was also performed in patient subgroups. All data was analyzed using SPSS 22.0 (SPSS, Chicago, IL).

## Results

### Baseline demographic characteristics

Prior to matching the baseline characteristics of the two study cohorts, the liver function profiles were shown to be significantly better in the TACE cohort than those in the control cohort (S1 Table).However, as shown in Table 1, the baseline demographic characteristics of the two groups were similar after patient matching. The median age of patients was 61 years old, and around 80% of patients were males. Nearly 90% of patients were infected with hepatitis virus B or C, and more than 40% of patients suffered from liver decompensation (Child-Pugh class B). In addition, the tumor status was quite severe in both groups. Most (> 50%) patients had extrahepatic metastases, while portal vein thrombosis could be found in more than 70% of patients. An AFP elevation > 400ng/ml was also common (~50%), and approximately 60% of patients suffered from a tumor morphology extension > 50% of the liver. The median point of the Cancer of the Liver Italian Program (CLIP) score was found to be as high as 3.

**Table 1:** Baseline characteristics of the study subjects.

Variables	Control Group (n = 28)	TACE Group (n = 28)	*p*
Age, year	60.5 (50.8–70.8)	60.9 (50.0–70.9)	0.25
Gender, n(%)			0.69
Male	22 (78.6%)	24 (85.7%)	
Female	6 (21.4%)	4 (14.3%)	
Etiology of disease, n (%)			0.99
Viral	25 (89.3%)	24 (85.7%)	
Non-viral	3 (10.7%)	4 (14.3%)	
Child-Pugh class, n (%)			0.99
A	15(53.6%)	16 (57.1%)	
B	13(46.4%)	12 (42.9%)	
Extrahepatic metastases, n (%)			0.77
No	13 (46.4%)	11 (39.3%)	
Yes	15 (53.6%)	17 (60.7%)	
Portal vein thrombosis, n (%)			0.75
No	8 (28.6%)	6 (21.4%)	
Yes	20 (71.4%)	22 (78.6%)	
AFP, ng/mL			0.79
> 400	14 (50.0%)	16 (57.1%)	
≤ 400	14 (50.0%)	12 (42.9%)	
Tumor morphology, n (%)			0.77
Extension ≤ 50%	12 (42.9%)	10 (35.7%)	
Extension > 50%	16 (57.1%)	18 (64.3%)	
CLIP score, point	3 (2–4)	3 (2–4)	1.00
Tx-naive before sorafenib therapy			0.38
Yes	4 (14.3%)	1 (3.6%)	
No	24 (85.7%)	27 (96.4%)	
Sorafenib therapy			
Initial daily dose, mg	400 (400–800)	400 (400–800)	0.45
Maximum daily dose, mg	800 (400–800)	400 (400–800)	0.45
Therapy duration, day	80 (42–243)	77 (42–169)	0.66
Reasons to stop sorafenib, n (%)			0.38
Disease progression	24 (85.7%)	27 (96.4%)	
Intolerance to side effect	4 (14.3%)	1 (3.6%)	
TACE course, number		2 (1–3)	
Time to the first TACE, day		34 (11.3–63.0)	

Note—Data of continuous variables are presented as median values (interquartile range). AFP = alpha-fetoprotein, CLIP = the Cancer of the Liver Italian Program, Tx = treatment

Before initiating sorafenib therapy, the majority of patients were not treatment-naïve (patient conditions prior to the initiation of sorafenib therapy are compared between the two study groups in S2 Table, and the patient characteristics remained similar between the two study groups). During sorafenib therapy, the median daily dose of sorafenib initiation was 400mg in both groups. Although the median maximum daily dose of sorafenib therapy was 800mg and 400mg daily in the control group and the TACE group respectively, the difference was not statistically significant. The median duration of sorafenib therapy was approximately 11 weeks in both groups, and more than 85% of patients stopped sorafenib therapy due to disease progression. In the TACE group, the median number of TACE course that patients received was 2 times, and the median duration from stopping sorafenib therapy to the first TACE was 34 days.

### Overall survival

**Fig 2:**
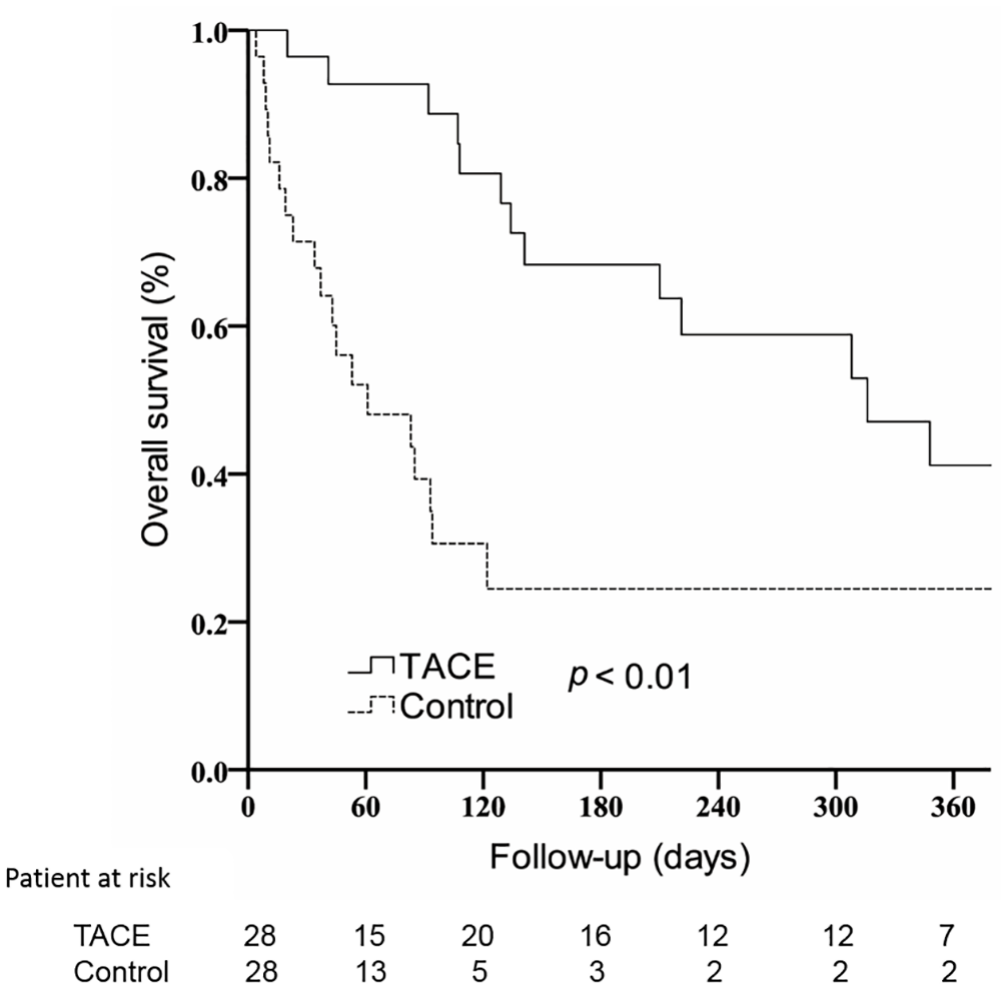
One-year overall survival in both the TACE and control groups after the follow-up index date.

Beforematching the follow-up index dateof the two groups in order to eliminateany immortal time bias, the median duration of patient survival in the TACE group was shown to be significantly longer than that of the control group (11.7 months, 95% CI: 6.0–17.4 months vs. 3.8 months, 95% CI: 2.1–5.4 months; *p* < 0.01) (S1 Fig).After patient matching was performed, as shown in Fig 2, the 1-year survival rate in the TACE group remainedsignificantly higher than that in the control group, after termination of sorafenib therapy (41.2%, 95% confidence interval [CI]: 19.4–63.0% vs. 24.5%, 95% CI: 6.3–42.7%; *p* < 0.01). In addition, the median duration of patient survival in the TACE group was significantly longer than that of the control group (7.2 months, 95% CI: 5.4–15.7 months vs. 1.6 months, 95% CI: 0.1–4.0 months; *p* < 0.01).

### Multivariate analysis of prognostic factors

As shown in Table 2, in univariate analysis, we initially found several potential prognostic factors (*p* <0.10), including TACE treatment, AFP > 400ng/mL, Child-Pugh class, and tumor extension > 50% of the liver. After being adjusted by AFP > 400ng/mL, Child-Pugh class B, and tumor extension > 50% of the liver, TACE treatment was shown to be an independent prognostic factor associated with a decreased risk of mortality (HR: 0.19; 95% CI: 0.08–0.42; *p* < 0.01). In contrast, tumor extension > 50% of the liver was considered to be an independent prognostic factor associated with an increased risk of mortality (HR: 2.99, 95% CI: 1.31–6.82; *p*< 0.01). Other potential prognostic factors, such as age, gender, serum AFP levels, Child-Pugh class B, extrahepatic metastases, portal vein thrombosis, HCC treatment before sorafenib therapy, and stopping sorafenib therapy due to side effect intolerance, were not found to be significantly associated with patient mortality in this study.

**Table 2:** Multivariate analysis for overall survival.

Variable	Univariate analysis			Multivariate analysis		
	HR	95% CI	*p*	HR	95% CI	*p*
TACE treatment	0.27	0.14–0.55	< 0.01	0.19	0.08–0.42	< 0.01
Age > 60 years	1.26	0.64–2.48	0.50			
Male gender	0.73	0.30–1.78	0.49			
Child-Pugh class B	1.94	0.99–3.78	0.05	1.72	0.86–3.43	0.13
Extrahepatic metastasis	1.59	0.78–3.24	0.20			
Portal vein thrombosis	1.15	0.50–2.65	0.74			
Tx-naïve before sorafenib therapy	1.45	0.44–4.83	0.54			
AFP > 400ng/mL	1.80	0.92–3.52	0.08	1.41	0.71–2.81	0.33
Tumor extension > 50%	1.87	0.91–3.84	0.09	2.99	1.31–6.82	0.01
Stopping sorafenib due to side effect intolerance	0.76	0.35–1.65	0.49			

Note—HR = Hazard ratio, CI = confidence interval, AFP = alpha-fetoprotein

### Stratified analysis for TACE treatment

**Fig 3:**
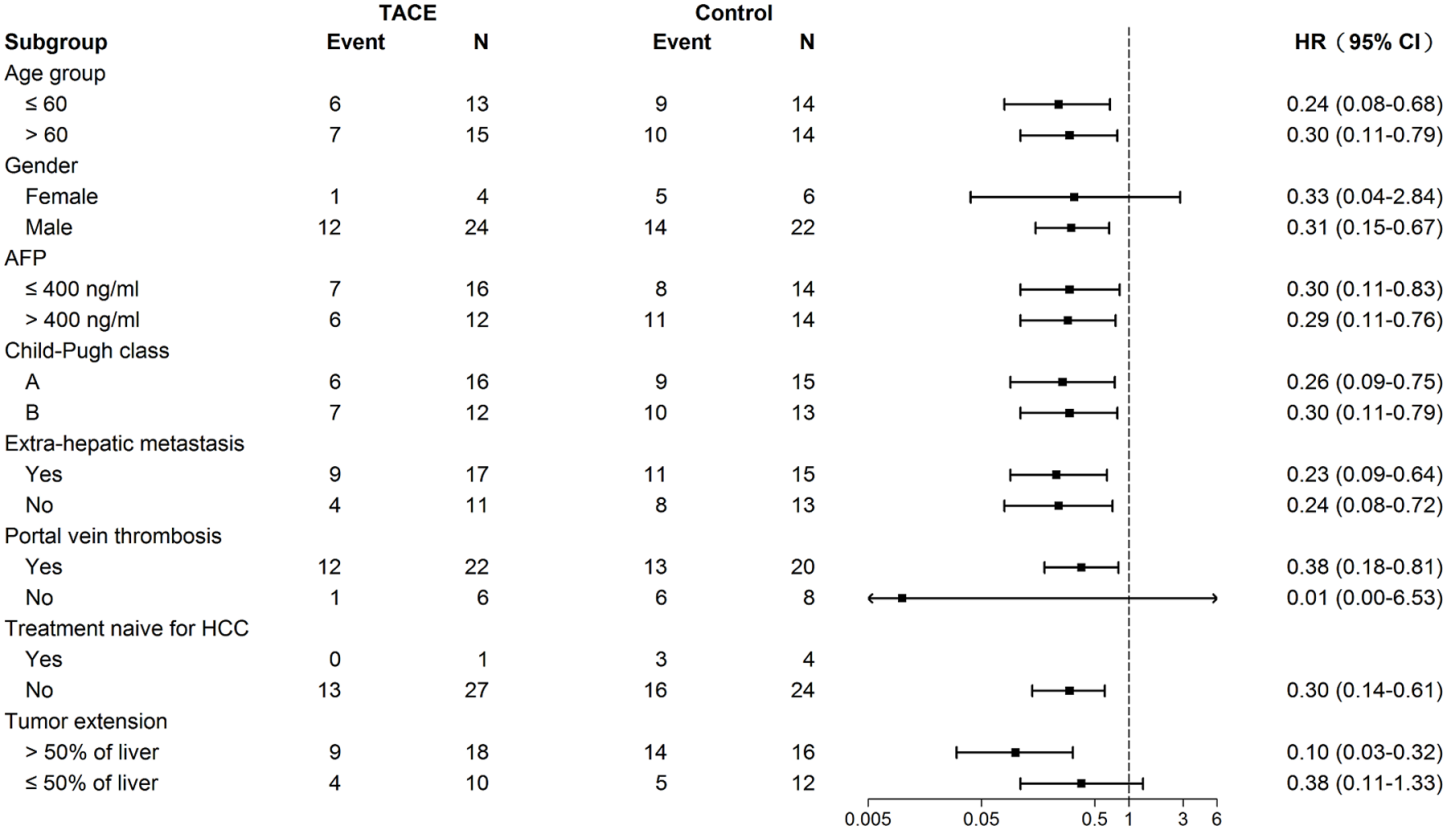
Stratified analysis in patient subgroups.

As shown in Fig 3, multivariate stratified analysis verified that TACE treatment was associated with a decreased mortality in each subgroup (all HR < 1.0). Furthermore, statistical significance was reached in most patient subgroups, including both age ≤ 60 and > 60 years, male gender, both AFP > 400ng/ml and ≤400 ng/ml, both Child-Pugh A and Child-Pugh B, with and without extrahepatic metastasis, with and without portal vein thrombosis, not treatment-naïve prior to sorafenib therapy, and tumor extension > 50% of the liver.

### Adverse events of TACE

The laboratory parameters before and after the first TACE treatment have been listed in S3a Table, where the changes in laboratory parameters were shown to be insignificant. In addition, the rates of TACE-related adverse effects have been listed in S3b Table, where most adverse effects were transient in nature. In this study, although as many as 70% of patients had portal vein thrombosis, the rates of TACE-related adverse effects were not significantly different between the patient groups who did or didn’t experience portal vein thrombosis (S3c Table). Similar results could also be noted between the patient groups in Child-Pugh class A and B (S3d Table). Although only one (3.6%) patient with portal vein thrombosis (in Child-Pugh A) died of TACE-related liver failure, liver decompensation in patients(14.3%) remained an adverse effect that should be given much attention. However, analyzing for all causes of mortality showed that TACE did not increase the liver failure-related mortality rate when compared to those in the control group (S4 Table).

## Discussion

Although TACE has been proven to be an effective treatment for locoregional HCC, the role of TACE treatment for advanced HCC after stopping sorafenib therapy remains unclear. To the best of our knowledge, this study was the first to reveal that TACE was shown to be an independent prognostic factor associated with decreased mortality risk after stopping sorafenib therapy. The findings of this study supported the theory that TACE treatment could be beneficial for select patients, however further prospective research remains mandatory for confirmation of our findings.

Upon failure of sorafenib therapy, the prognosis for patients with advanced HCC is generally poor, with a median survival period of only 4–8 months, as shown in previous studies [7,14]. These poor outcome results could be due to rapid tumor growth in the liver [7,8], with patient survival being possibly improved by means of controlling intrahepatic tumor growth. Although portal vein thrombosis has been traditionally considered a relative contraindication to TACE, recent studies have shown that TACE treatment could be safe due to the development of collateral circulation [4,13,25]. In patients experiencing portal vein thrombosis, TACE treatment may increase survival rates by 15–25% over a year [26–29], with a gain of 3–6 months over the median survival time [30]. In a recent meta-analysis of TACE for 1,933 HCC patients with main portal vein thrombosis, only 1% experienced liver failure, indicating that main portal vein thrombosis should not be considered as an absolute contraindication to TACE [31]. In this study, as many as 70% of patients experienced portal vein thrombosis, but the findings of this study still suggest that TACE could be beneficial in select patients with portal vein thrombosis.

According to the multivariable analysis in this study, tumor extension > 50% of the liver is another independent prognostic factor related to patient mortality, where a large tumor burden may reduce the tumor suppression effect of TACE. However, TACE was still beneficial for patients with large liver tumors in the subgroup analysis. Furthermore, in patients experiencing extrahepatic metastasis, as many as 80% of those patients may die of intrahepatic tumor progression [19,20]. Previous studies have suggested that TACE could be beneficial for survival in patients with extrahepatic metastasis, whilst controlling their intrahepatic tumors may thus still further improve patient survival [20,32]. As the findings in this study show, although approximately half of the patients had extrahepatic metastasis, TACE was still associated with a better patient survival rate during subgroup analysis.

Regorafenib is currently the only second-line treatment in phase 3 trials demonstrated to be survival-beneficial in patients with advanced HCC [14]. However, the median survival period in the regorafenib group was increased by only 2.8 months (10.6 months vs. 7.8 months), with a response rate of 11% in the regorafenib group. In addition, as many as 40% of patients in the regorafenib group could not tolerate the adverse effects and therefore discontinued regorafenib. Furthermore, regorafenib may not be affordable in most countries, whilst TACE remains a commonplace treatment worldwide. Although regorafenib may become standard therapy after the occurrence of sorafenib failure, other treatment choices should also be welcomed. According to the findings in this study, TACE could be a treatment option after the stopping of sorafenib therapy for select patients.

Several limitations in this study should be mentioned. First, selection bias might exist in this retrospective study; e.g., clinicians may tend to suggest TACE treatment for patients who have a better life expectancy. However, in this study, important prognostic factors, such as Child-Pugh class and tumor burden, were well matched, while patients with severe liver decompensation (Child-Pugh class C), or poor performance status were excluded. Furthermore, in order to avoid any immortal time bias, the index date of outcome follow-up in the control group was matched with those in the TACE group. The selection bias should have been minimized. However, a well-controlled prospective study is important to confirm our findings. Second, the sample size of this study was relatively small, and the statistical analysis numbers for some patient subgroups might be undermanned. However, our study can still provide a clinically useful direction, while we realize that further studies with larger sample sizes should be encouraged. Third, although TACE was found to be effective in improving patient survival rates in this study, not all patients were suitable for TACE treatment after they stopped sorafenib therapy. Therefore, the rules for selecting patients for TACE should be further developed in the future.

In summary, TACE treatment after stopping sorafenib therapy for advanced HCC was found to be associated with a decreased mortality risk in this study. Therefore, TACE could be considered as a second-line treatment option.

## Supporting information

S1 Fig The 1-year survival rate in the two study groups before index date matching. (DOCX)

S1 Table Baseline characteristics of the study subjects before patient matching. (DOCX)

S2 Table Baseline characteristics of the study subjects before the initiation of sorafenib therapy. (DOCX)

S3 Table The analysis on the adverse effects of TACE treatment (a) The laboratory parameters before and after the first TACE treatment; (b) The rates of TACE-related adverse effects; (c) The rates of TACE-related adverse effects in the patient groups with or without portal vein thrombosis. (DOCX)

S4 Table All causes of mortality for the study subjects. (DOCX)
