# Supplementary figures and images for: Transcatheter arterial chemoembolization after stopping sorafenib therapy for advanced hepatocellular carcinoma
Source: PLoS One. 2017 Nov 30;12(11):e0188999. doi: 10.1371/journal.pone.0188999 (PMC5708733; doi:10.1371/journal.pone.0188999)

S1 Fig. The 1-year survival rate in the two study groups before index date matching.


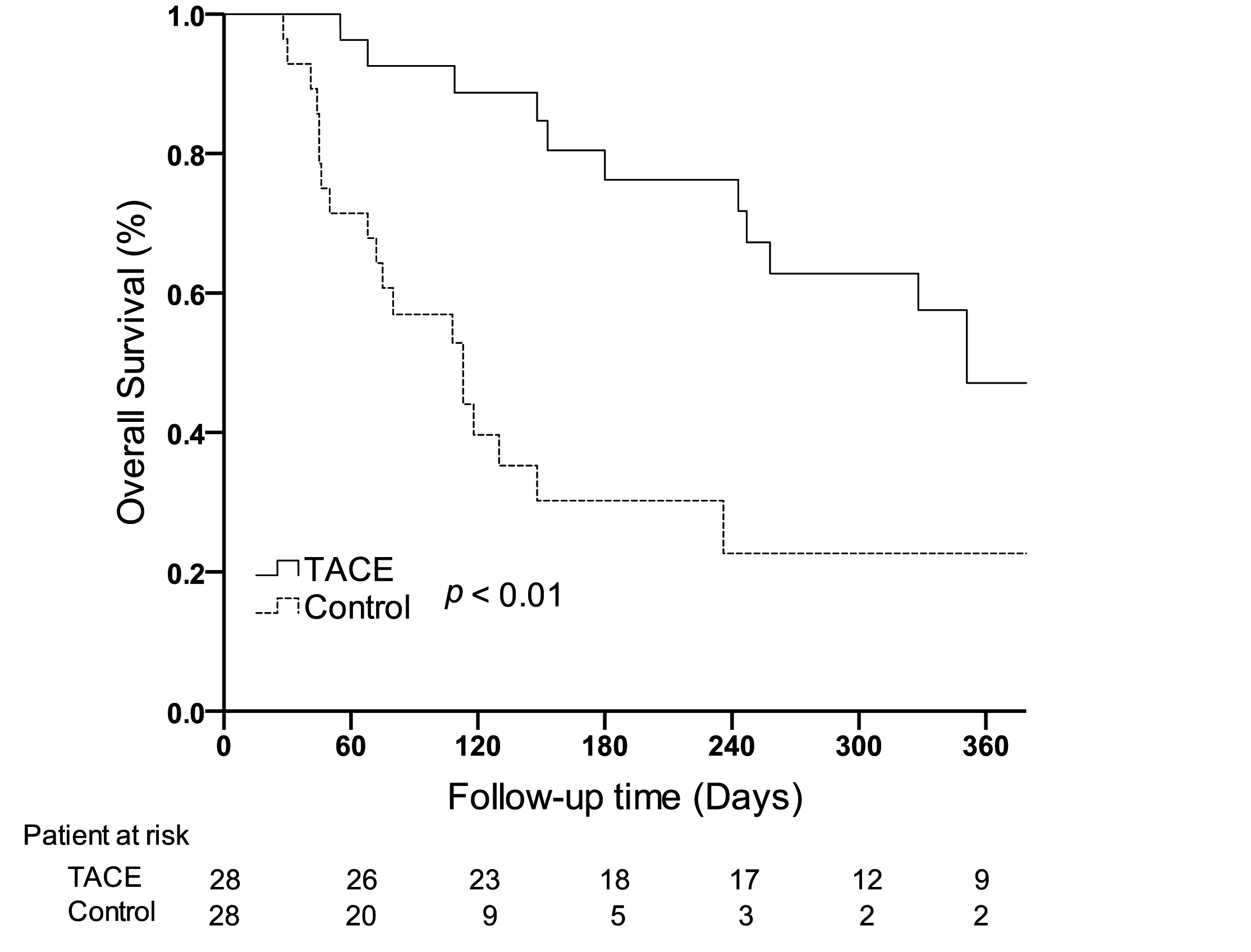

Supplement: S1 Fig — (DOCX) [file pone.0188999.s001.docx]
