# Supplementary material for: Transcatheter arterial chemoembolization after stopping sorafenib therapy for advanced hepatocellular carcinoma
Source: PLoS One. 2017 Nov 30;12(11):e0188999. doi: 10.1371/journal.pone.0188999 (PMC5708733; doi:10.1371/journal.pone.0188999)
Supplement: S1 Table — (DOCX) [file pone.0188999.s002.docx]

S1 Table. Baseline characteristics of the study subjects before patient matching

| Variables | Control Group  (n = 91) | | TACE Group  (n = 35) | *p* |  |
| --- | --- | --- | --- | --- | --- |
| Age, year | 58.0 (50.0-64.0) | | 60.6 (50.1-71.0) | 0.34 |  |
| Gender, n (%) |  | |  | 0.99 |  |
| Male | 77 (84.6%) | | 30 (85.7%) |  |  |
| Female | 14 (15.4%) | | 5 (14.3%) |  |  |
| Etiology of disease, n (%) |  | |  | 0.99 |  |
| Viral | 81 (89.0%) | | 31 (88.6%) |  |  |
| Non-viral | 10 (11.0%) | | 4 (11.4%) |  |  |
| Child-Pugh class, n (%) |  | |  | 0.29 |  |
| A | 38 (41.8%) | | 19 (54.3%) |  |  |
| B | 53 (58.2%) | | 16 (45.7%) |  |  |
| Extrahepatic metastases, n (%) |  | |  | 0.89 |  |
| No | 42 (46.2%) | | 15 (42.9%) |  |  |
| Yes | 49 (53.8%) | | 20 (57.1%) |  |  |
| Portal vein thrombosis, n (%) |  | |  | 0.45 |  |
| No | 26 (28.6%) | | 7 (20.0%) |  |  |
| Yes | 65 (71.4%) | | 28 (80.0%) |  |  |
| AFP, ng/mL |  | |  | 0.13 |  |
| > 400 | 37 (40.7%) | | 20 (57.1%) |  |  |
| ≤ 400 | 54 (59.3%) | | 15 (42.9%) |  |  |
| Tumor morphology, n (%) |  | |  | 0.99 |  |
| Extension ≤ 50% | 27 (29.7%) | | 10 (28.6%) |  |  |
| Extension > 50% | 64 (70.3%) | | 25 (71.4%)) |  |  |
| CLIP score, point | 4.0 (3.0, 5.0) | | 3.0 (2.0, 4.0) | 0.06 |  |
| Tx-naive before sorafenib therapy | |  |  | 0.16 |  |
| Yes | 16 (17.6%) | | 2 (5.7%) |  |  |
| No | 75 (82.4%) | | 33 (94.3%) |  |  |
| Sorafenib therapy |  | |  |  |  |
| Initial daily dose, mg | 400 (400- 800) | | 400 (400-800) | 0.72 |  |
| Maximum daily dose, mg | 800 (400-800) | | 400 (400-800) | 0.53 |  |
| Therapy duration, day | 70 (41-179) | | 102 (42-201) | 0.37 |  |
| Reason to stop sorafenib, n (%) |  | |  | 0.05 |  |
| Disease progression | 76 (83.5%) | | 23 (65.7%) |  |  |
| Intolerance to side effect | 15 (16.5%) | | 12 (34.3%) |  |  |
| TACE course, number |  | | 2 (1-3) |  |  |
| ALT, U/L | 57.0 (36.0-117.0) | | 51.0 (35.0-98.0) | 0.44 |  |
| Total bilirubin, mg/dL | 1.5 (0.9-2.6) | | 0.9 (0.6-1.3) | <0.01 |  |
| Albumin, g/dL | 3.2 (2.9-3.6) | | 3.7 (3.2-3.9) | <0.01 |  |
| Prothrombin time (INR) | 1.2 (1.1-1.3) | | 1.1 (1.0-1.2) | <0.01 |  |

Note－Data of continuous variables are presented as median values (interquartile range). AFP = alpha-fetoprotein, CLIP = the Cancer of the Liver Italian Program, Tx = treatment, ALT= alanine transaminase, INR=international normalized ratio
