# Supplementary material for: Transcatheter arterial chemoembolization after stopping sorafenib therapy for advanced hepatocellular carcinoma
Source: PLoS One. 2017 Nov 30;12(11):e0188999. doi: 10.1371/journal.pone.0188999 (PMC5708733; doi:10.1371/journal.pone.0188999)
Supplement: S2 Table — (DOCX) [file pone.0188999.s003.docx]

S2 Table. Baseline characteristics of the study subjects before the initiation of sorafenib therapy

| Variables | Control Group  (n = 28) | | | | | TACE Group  (n = 28) | *p* |  |
| --- | --- | --- | --- | --- | --- | --- | --- | --- |
| The timing of HCCdiagnosis, n (%) | |  | | | |  | 0.59 |  |
| First study | 13 (46.4%) | | | | | 16 (57.1%) |  |  |
| Surveillance protocol | 15 (53.6%) | | | | | 12 (42.9%) |  |  |
| Method of diagnosis, n (%) |  | | | | |  |  |  |
| Histopathology | 2 (7.1%) | | | | | 1 (3.6%) | 0.99 |  |
| Image study | 26 (92.9%) | | | | | 27 (96.4%) |  |  |
| LIRADS image score, n (%) |  | | | | |  | 0.50 |  |
| LR-4 | 15 (57.7%) | | | | | 19 (70.4%) |  |  |
| LR-5 | 11 (42.3%) | | | | | 8 (29.6%) |  |  |
| Treatment before sorafenib therapy, n (%) | | |  | | |  |  |  |
| Surgery | 10 (35.7%) | | | | | 11 (39.3%) | 0.99 |  |
| Local ablation | 0 (0.0%) | | | | | 3 (10.7%) | 0.24 |  |
| TACE | 8 (28.6%) | | | | | 14 (50.0%) | 0.17 |  |
| Others | 6 (21.4%) | | | | | 6 (21.4%) | 1.00 |  |
| Degree of tumor invasion, n (%) | | | |  | |  | 0.86 |  |
| Extrahepactic metastases, | 11 (39.3%) | | | | | 10 (35.7%) |  |  |
| Portal vein thrombosis | 12 (42.9%) | | | | | 14 (50.0%)) |  | |
| Both | 5 (17.9%) | | | | | 4 (14.3%) |  |  |
| Tumor morphology in liver, n (%) | | | |  | |  | 0.59 |  |
| Extension ≤ 50% | 18 (64.3%) | | | | | 15 (53.6%) |  |  |
| Extension > 50% | 10 (35.7%) | | | | | 13 (46.4%) |  |  |
| Child-Pugh class, n (%) |  | | | | |  | 1.00 |  |
| A | 24 (85.7%) | | | | | 24 (85.7%) |  |  |
| B | 4 (14.3%) | | | | | 4 (14.3%) |  |  |
| ALT, U/L | 43.0 (28.8-113.8) | | | | | 52.0 (34.0-89.3) | 0.53 |  |
| Albumin, g/dL | 3.7 (2.9-3.9) | | | | | 3.9 (3.3-4.0) | 0.15 |  |
| Total bilirubin, mg/dL | 1.0 (0.6-1.4) | | | | | 0.8 (0.6-1.3) | 0.38 |  |
| Prothrombin time, INR | 1.07 (1.04-1.11) | | | | | 1.06 (1.00-1.13) | 0.43 |  |
| Concomitant treatment with sorafenib, n (%) | | | | |  |  |  |  |
| TACE | 8 (28.6%) | | | | | 14 (50.0%) | 0.10 |  |
| Radiotherapy | 6 (21.4%) | | | | | 6 (21.4%) | 1.00 |  |
| Others | 0 (0.0%) | | | | | 0 (0.0%) | - |  |

Note－ LI-RADS= Liver Imaging Reporting and Data Systemby The American College of Radiology
