# Supplementary material for: Transcatheter arterial chemoembolization after stopping sorafenib therapy for advanced hepatocellular carcinoma
Source: PLoS One. 2017 Nov 30;12(11):e0188999. doi: 10.1371/journal.pone.0188999 (PMC5708733; doi:10.1371/journal.pone.0188999)
Supplement: S3 Table — (DOCX) [file pone.0188999.s004.docx]

S3 Tables. The analysis on the adverse effects of TACE treatment

S3a Table. The laboratory parameters before and after the first TACE treatment

| Variables | Before  (n = 28) | After TACE  (n = 28) | *p* |
| --- | --- | --- | --- |
| ALT, U/L | 52.0 (38.0-107.3) | 79.5 (33.5-187.8) | 0.10 |
| Total bilirubin, mg/dL | 1.0 (0.6-1.5) | 1.0 (0.7-2.3) | 0.30 |
| Albumin, g/dL | 3.8 (3.1-4.0) | 3.5 (3.1-3.7) | 0.25 |
| Prothrombin time, INR | 1.06 (1.03-1.15) | 1.07 (1.04-1.15) | 0.32 |

Note－TACE= Transcatheter Arterial Chemoembolization, ALT= Alanine aminotransferase

S3b Table. The rates of TACE-related adverse effects

| Variables | Patients receiving TACE  (n = 28) |
| --- | --- |
| Fever, n (%) | 6 (21.4%) |
| Abdominal pain, n (%) | 3 (10.7%) |
| Nausea and/or vomiting, n (%) | 2 (7.1%) |
| Catheterization-site bleeding, n (%) | 0 (0%) |
| Liver decompensation, n (%) | 4 (14.3%) |
| Transient liver decompensation, n (%) | 2 (7.1%) |
| TACE-related mortality, n (%) | 1 (3.6%) |
| Varices bleeding, n (%) | 0 (0%) |

Note－ TACE-related mortality defined as patient died of TACE-related complications within 1 month after the TACE procedure. Liver decompensation defined as doubling of bilirubin level, new onset of ascites or hepaticencephalopathy, or changes of Child-Pugh classification within two weeks after the TACE procedure.

TACE= Transcatheter Arterial Chemoembolization

S3c Table. The rates of TACE-related adverse effects in the patient groups with or without portal vein thrombosis

| Variables | PVT  (n = 19) | No PVT  (n = 13) | *p* |
| --- | --- | --- | --- |
| Fever, n (%) | 2 (9.1%) | 4 (66.7%) | 0.58 |
| Abdominal pain, n (%) | 3 (13.6%) | 1 (16.7%) | 0.99 |
| Nausea and/or vomiting, n (%) | 2 (9.1%) | 0 (0.0%) | 0.99 |
| Catheterization-site bleeding, n (%) | 0 (0.0%) | 0 (0.0%) | - |
| Liver decompensation, n (%) | 4 (18.2%) | 0 (0.0%) | 0.54 |
| Transient Liver decompensation, n (%) | 2 (9.1%) | 0 (0.0%) | 0.99 |
| TACE related mortality, n (%) | 1 (4.5%) | 0 (0.0%) | 0.99 |
| Varices bleeding, n (%) | 0 (0.0%) | 0 (0.0%) | - |

Note－PVT= portal vein thrombosis

S3d Table. The rates of TACE-related adverse effects in the patient groups in Child-Pugh class A or B

| Variables | Child-Pugh A  (n=16) | Child-Pugh B  (n = 12) | *p* |
| --- | --- | --- | --- |
| Fever, n (%) | 3 (18.8%) | 3 (25.0%) | 0.99 |
| Abdominal pain, n (%) | 1 (6.3%) | 3 (25.0%) | 0.29 |
| Nausea and/or vomiting, n (%) | 2 (12.5%) | 0 (0.0%) | 0.49 |
| Catheterization-site bleeding, n (%) | 0 (0.0%) | 0 (0.0%) | - |
| Liver decompensation, n (%) | 2 (12.5%) | 2 (16.7%) | 0.99 |
| Transient Liver decompensation, n (%) | 1 (6.3%) | 1 (8.3%) | 0.99 |
| TACE-related mortality, n (%) | 1 (6.3%) | 0 (0.0%) | 0.99 |
| Varices bleeding, n (%) | 0 (0.0%) | 0 (0.0%) | - |
