# Supplementary material for: Transcatheter arterial chemoembolization after stopping sorafenib therapy for advanced hepatocellular carcinoma
Source: PLoS One. 2017 Nov 30;12(11):e0188999. doi: 10.1371/journal.pone.0188999 (PMC5708733; doi:10.1371/journal.pone.0188999)
Supplement: S4 Table — (DOCX) [file pone.0188999.s005.docx]

S4 Table. All causes of mortality for the study subjects

| Variables | Control group  (n = 28) | TACE group  (n = 28) | *p* |
| --- | --- | --- | --- |
| Liver failure, n (%) | 8 (28.6%) | 6 (21.4%) | 0.57 |
| TACE-related liver failure, n (%) | 0 (0.0%) | 1 (3.6%) |  |
| Gastrointestinal bleeding, n (%) | 5 (17.9%) | 2 (7.1%) |  |
| Acute cholangitis, n (%) | 2 (7.1%) | 1 (3.6%) |  |
| Respiratory failure, n (%) | 2 (7.1%) | 1 (3.6%) |  |
| Tumor rupture, n (%) | 1 (3.6%) | 1 (3.6%) |  |
| Cerebral infarction, n (%) | 0 (0.0%) | 1 (3.6%) |  |
| Spontaneous bacterial peritonitis, n (%) | 1 (3.6%) | 0 (0.0%) |  |
